# Phylogenetic and genomic insights into magnetosome biomineralization in magnetotactic *Alphaproteobacteria*

**DOI:** 10.1128/aem.02121-25

**Published:** 2025-11-28

**Authors:** Rongrong Zhang, Peiyu Liu, Jinling Bai, Kelei Zhu, Yan Liu, Andrew P. Roberts, Yongxin Pan, Jinhua Li

**Affiliations:** 1 Key Laboratory of Deep Petroleum Intelligent Exploration and Development, Institute of Geology and Geophysics, Chinese Academy of Sciences 66451, Beijing, China; 2 Laboratory for Marine Geology, Qingdao Marine Science and Technology Center 554912 , Qingdao, China; 3 Southern Marine Science and Engineering Guangdong Laboratory 590852 , Zhuhai, China; 4 College of Earth and Planetary Sciences, University of Chinese Academy of Sciences 105734, Beijing, China; 5 Research School of Earth Sciences, Australian National University 2219, Canberra, Australia; 6 Key Laboratory of Earth and Planetary Physics, Institute of Geology and Geophysics, Innovation Academy for Earth Science, Chinese Academy of Sciences 85415 , Beijing, China; Colorado School of Mines, Golden, Colorado, USA

**Keywords:** horizontal gene transfer, phylogenomics, *Alphaproteobacteria*, magnetosome biomineralization, magnetotactic bacteria

## Abstract

Magnetotactic bacteria (MTB) biomineralize intracellular, membrane-enclosed magnetite or greigite nanocrystals (magnetosomes). How magnetosome gene clusters (MGCs) control magnetosome morphology and evolve across lineages remains central to reconstructing the history of magnetotaxis. Here, we report five uncultured MTB strains from Yuyuantan Lake (Beijing, China), all within *Rhodospirillales* order (*Alphaproteobacteria* class). Using phylogenetics, fluorescence *in situ* hybridization-scanning electron microscopy, and transmission electron microscopy, we show that magnetosome morphology is more strongly constrained by phylogeny than by cell morphology. Whole-genome comparisons and MGC phylogenies indicate that vertical inheritance predominates at the genus level, whereas topological incongruences reveal additional processes, notably horizontal transfer and gene duplication. In particular, the presence of a canonical *mamAB* operon together with a duplicated *mamAB-2* cluster supports inter-genus horizontal gene transfer between *Magnetospirillum* and *Paramagnetospirillum*. These findings refine evolutionary models by showing that conserved MGC architectures provide a stable scaffold for magnetosome biomineralization while permitting diversification within the *Alphaproteobacteria* class.

**IMPORTANCE:**

Magnetotactic bacteria (MTB) build intracellular magnetic nanoparticles (magnetosomes) that guide navigation and influence biogeochemical cycling. Yet how the underlying genes map onto ancestry and crystal shape remains unclear. Pairing quantitative crystal-morphology statistics with phylogenomic analysis for MTB from the *Rhodospirillales* order, we show that magnetosome traits carry a stronger phylogenetic signal than cell shape. Newly recovered uncultured strains broaden *Paramagnetospirillum* diversity, and a high-quality genome (YYTV-2) represents a novel species within the rarely studied *Candidatus* Magneticavibrio. Analyses of both the canonical *mamAB* operon and a duplicated *mamAB-2* cluster indicate predominantly vertical inheritance, with horizontal transfer and gene duplication introducing modular variation. These results tighten genotype-mineral phenotype links, improving the interpretation of magnetofossils and MTB as indicators of environmental change.

## INTRODUCTION

Magnetotactic bacteria (MTB) biomineralize intracellular, membrane-enclosed nanocrystals of magnetite (Fe_3_O_4_) or greigite (Fe_3_S_4_), termed magnetosomes (1–4). Magnetosomes are commonly arranged into chains that act as a cellular biocompass, aligning cells with geomagnetic field lines and facilitating efficient migration to microoxic environments (5–8). By shuttling along redox gradients, MTB influence biogeochemical cycling of C, N, S, P, Fe, and Si in aquatic oxic-anoxic transition zones (9–13). Beyond their ecological roles, MTB also generate sedimentary magnetofossils that archive paleomagnetic and paleoenvironmental information (14–16).

Within bacteria, members of the order *Rhodospirillales* in the *Alphaproteobacteria* class include the most cultivated MTB (17–19) and exhibit broad diversity in both magnetosome gene clusters (MGCs) and crystal morphologies (20, 21). Genera include *Magnetospirillum*, *Paramagnetospirillum*, *Ca*. Magneticavibrio, *Magnetospira*, *Magnetovibrio*, and *Terasakiella* (22–24). Two dominant crystal habits, cubo-octahedral and prismatic, are observed across these taxa (21, 25, 26). Despite morphological diversity, *Rhodospirillales* MTB share a conserved *mamAB* operon (e.g., *mamA*, *mamB*, *mamM*, *mamQ*, *mamO*, *mamK*, and *mamE*) (20, 27). In contrast, the *mms6* cluster (*mms48*, *mms36*, *mmsF*, and *mms6*) is conserved mainly in *Magnetospirillum*, *Paramagnetospirillum*, and *Ca*. Magneticavibrio (28, 29), whereas *mamXYZ* and *mamCDFG*, typically restricted to *Magnetospirillum* and *Paramagnetospirillum*, occur at the margins of *mamAB* or elsewhere in the genome (30, 31).

How MGC evolution controls magnetosome traits remains a central question (19, 20). Early phylogenetic analyses of *mam* genes pointed to predominantly vertical inheritance (32, 33), with later work revealing exceptions involving intra-genus horizontal gene transfer (HGT) and repeated acquisitions (27). More recently, gene duplication, including *mamAB*-like operons, has emerged as an additional driver of innovation (19, 34). Taken together, these observations support a mixed model in which vertical inheritance predominates but HGT, duplication, and gene loss also shape MGC diversity (19). However, most studies have focused on the canonical *mamAB* operon, leaving the dynamics and evolutionary roles of duplicated clusters (e.g., *mamAB-2*), and their potential for cross-lineage exchange, insufficiently resolved (20, 27).

In this study, we characterize five uncultured *Rhodospirillales* MTB from Yuyuantan Lake (Beijing, China), including a vibrioid strain (YYTV-2) with a high-quality genome assigned to the rarely studied *Ca*. Magneticavibrio genus. We couple quantitative morphology with phylogenomics to test how variation in MGC organization relates to magnetosome biomineralization. By explicitly analyzing both the canonical *mamAB* and the duplicated *mamAB-2* clusters, we assess the relative contribution of vertical inheritance, gene duplication, and HGT to MGC evolution within *Rhodospirillales*, especially in *Magnetospirillum* and *Paramagnetospirillum*, thereby refining current models of magnetotaxis diversification in *Alphaproteobacteria*.

## MATERIALS AND METHODS

### Field sampling, MTB collection, and sample preparation

Surface sediments were collected from Yuyuantan Lake (Beijing, China; 39°54′50.53″N, 116°18′32.98″E) in April 2022. *In situ* parameters, including salinity (≈0.30‰), pH (≈7.25), and temperature (~22.8°C), were measured with an HQ40d portable multiparameter meter (Hach, USA). Sediment and overlying water (depth 1–2 m) were transferred to 500 mL opaque, wide-mouth reagent bottles at 2:1 sediment:water ratio. Upon arrival, the bottle caps were loosened, and the samples were incubated in the dark at ~25°C to establish laboratory microcosms (35). After 2 weeks, MTB within the sediment was examined by the hanging-drop method under an optical microscope (36). Living MTB cells were extracted magnetically using modified capillary tubes (37), washed three times with Milli-Q water, and divided into triplicates for microscopic and molecular analyses (38).

### Primer evaluation and 16S rRNA gene sequencing

Primer coverage and specificity were assessed using the SILVA probe matching and evaluation tool TestProbe 3.0 (https://www.arb-silva.de/search/testprobe/) (39). To evaluate primer efficiency in amplifying 16S rRNA genes, we used the TestPrimer 1.0 software (https://www.arb-silva.de/search/testprime/) with the SILVA databases (40).

Polymerase chain reaction (PCR) amplification of MTB 16S rRNA genes was conducted separately using general bacterial primers (27F/1492R) (41) and alphaproteobacteria-specific primers (28F/1391R) (41, 42), following the method outlined by Li et al. (38). PCR products were purified using a QIAquick Gel Extraction Kit (Qiagen, Germany), ligated into the pMD19-T vector (TaKaRa, Japan), and cloned into *Escherichia coli* strain DH5α competent cells (Huada Genome Center, Beijing, China). For each sample, 20–50 clones were selected randomly and sequenced with vector primers M13F-47 and M13R-48 (Huada Genome Center, Beijing, China). Sequences shorter than 1,100 bp were discarded, and the remaining sequences were clustered into operational taxonomic units (OTUs) using the cd-hit software (43) with a 98.7% identity threshold (species criterion) (44). Sequence quality was checked and corrected using the Gblocks 0.9B online algorithm (45).

### FISH-SEM correlative analysis for MTB identification

Based on the OTUs of the MTB obtained (Table S1), five species-specific oligonucleotide probes were designed: YYTV2-920, YYTS2-1106, YYTS3-924, YYTS5-917, and YYTS7-532 (Table S2). Probe specificity (Table S3) was evaluated using the TestProbe 3.0 tool (39). The universal bacteria probe EUB338 (5′-GCTGCCTCCCGTAGGAGT-3′) (46) served as a positive control. Fluorescence *in situ* hybridization (FISH) was performed according to Li et al. (38), with species-specific probes labeled with Cy3 and EUB338 labeled with FAM. *E. coli* DH5α cells were used as internal controls. FISH samples were examined using an Olympus BX51 microscope equipped with phase-contrast and fluorescence optics, and images were captured with a DP70 digital camera system (Olympus Corp., Tokyo, Japan). For correlative analysis with scanning electron microscopy (SEM), samples were carbon-coated using a Leica ACE200 low-vacuum sputter coater (Leica Microsystems, Wetzlar, Germany) and were analyzed with a Zeiss Ultra-55 field emission SEM at a working voltage of 5 kV and a working distance of 5 mm.

### Genome sequencing, assembly, and binning of strain YYTV-2

Genomic DNA from living MTB cells was amplified using the REPLI-g Single Cell Kit (QIAGEN, Germany), following the manufacturer’s protocol. Amplified products were purified with the QIAEX II Gel Extraction Kit (QIAGEN, Germany) to remove short sequences and protein residues. The purified DNA was sequenced on the Illumina HiSeq 6000 system (Annoroad, Beijing, China) using a paired-end strategy (150 bp reads, average insert size of 270 bp).

Raw sequencing reads were processed to remove adapters and low-quality sequences using Trimmomatic software (47). The clean reads were assembled into contigs using IDBA software (48), with kmer values of 30, 60, 90, and 120. Optimal contigs were selected based on k-mer analysis. Genome binning and assembly refinement were performed using MetaWRAP software (49), with exclusion of contigs shorter than 1,500 bp. Genome completeness and contamination were assessed using the CheckM2 software (50), with genomes not meeting quality thresholds (completeness ≥ 70% and contamination ≤ 10%) being discarded. A 3.36 Mb draft genome for strain YYTV-2 was obtained, including a partial 16S rRNA gene sequence (641 bp) that showed 100% identity to the corresponding gene in strain YYTV-2.

Average nucleotide identity (ANI) values were calculated using the ANI calculator at EzBioCloud (https://www.ezbiocloud.net/) with default settings (51). Average amino acid identity (AAI) values were obtained using the AAI calculator in the Enveomics Gateway (https://enveomics.scigap.org/) (52, 53), with parameters set to minimum length 150 bp, minimum score 50, minimum alignments 50, and minimum identity 30%.

### Gene annotation and phylogenetic analyses

Gene annotation was performed using the Genemark online database (54). Homologous magnetosome proteins, including Mam-A, -B, -E, -I, -K, -M, -P, and -Q, were identified through BLAST searches against the refseq_protein database, using protein sequences from *Magnetospirillum gryphiswaldense* MSR-1 and *Ca*. Magneticavibrio boulderlitore LM-1 as queries. Proteins with ≥40% identity and ≥90% coverage were considered homologous. Additional magnetosome proteins and MGC proteins were validated using the NCBI BLAST web server (https://blast.ncbi.nlm.nih.gov/Blast.cgi).

A whole-genome phylogenetic tree was constructed using the genome data obtained here, along with other *Alphaproteobacteria* MTB genomes from the NCBI database (https://www.ncbi.nlm.nih.gov/). The “classify_wf” command in GTDB-Tk v.0.1.3 identified 120 single-copy bacterial marker protein sequences for alignment, and taxonomic assignments were made based on the GTDB r86 database (55). A maximum-likelihood tree was constructed using IQ-TREE (56), with evolutionary models selected by ModelFinder (57) and 1,000 ultrafast bootstraps. The phylogenetic trees of core MGC genes and 16S rRNA genes were generated similarly.

### Transmission electron microscopy (TEM) and statistical analysis

TEM and high-resolution TEM (HRTEM) observations, along with selected area electron diffraction (SAED) analyses, were conducted using a JEM2100 microscope (JEOL Ltd., Tokyo, Japan) at a working voltage of 200 kV. Particle size, number, and length and width of magnetite crystals were measured from TEM images using ImageJ software (https://imagej.nih.gov/ij/).

To explore the relationship between phylogeny and morphology (including cell size, shape, and magnetosome chain configuration), non-metric multidimensional scaling (nMDS) analysis was performed using the *vegan* package in R. This rank-based method substituted original distances with ranks for six physical features: cell length, cell width, crystal number, crystal morphology, crystal width, and axial ratio (width/length). Envfit analysis was used to overlay morphological features onto the nMDS ordination, determining the significance of each feature to the ordination.

## RESULTS

### Identification of *Alphaproteobacteria* MTB in Yuyuantan Lake sediments

Optical microscopic observations of two laboratory microcosms (hereafter termed microcosms 1 and 2) showed spirilla and vibrios as the dominant magnetotactic morphotypes (Fig. S1). Using modified capillary tubes (37), living MTB cells were magnetically isolated and their 16S rRNA genes were amplified for phylogenetic analysis. Initial PCR with the universal bacterial primer pair (27F/1492R) yielded 54 sequences, most showing 98.89–99.93% similarity to previously characterized magnetotactic cocci (Table S4).

To target magnetotactic *Alphaproteobacteria* more selectively, we repeated PCR with *Alphaproteobacteria*-specific primers (28F/1391R) (42). This offers higher *Alphaproteobacteria* coverage than the universal primers (41) and more effectively amplifies near-full-length 16S rRNA sequences than other *Alphaproteobacteria*-specific primers (58), with minimal cross-coverage across other phyla/classes (Table S5 and S6). This produced 49 16S rRNA gene sequences; roughly half corresponded to known magnetotactic cocci or non-MTB, and the remainder clustered into five OTUs (> 94.5% similarity to known magnetotactic *Alphaproteobacteria*; Table S1).

The five OTUs were: OTU1_28F_ (*n* = 9, hereafter YYTV-2), ~96.61% similar to *Ca*. Magneticavibrio boulderlitore LM-1 (Genbank accession: JF490044); OTU2_28F_ (*n* = 8, YYTS-2) is ~98.38% similar to uncultured MTB clone WYH-50 (Genbank accession: JX537788); OTU3_28F_ (*n* = 2, YYTS-3) is ~95.32% similar to *Paramagnetospirillum* sp. B9-5-1 (Genbank accession: FJ562217); OTU7_28F_ (*n* = 3, YYTS-5) and OTU8_28F_ (*n* = 2, YYTS-7) show ~96.75% and ~94.60% similarity, respectively, to *Paramagnetospirillum marisnigri* SP-1 (Genbank accession: NR149242). By the 98.7% species delineation threshold (44), these lineages represent novel *Alphaproteobacteria* species.

**Fig 1:**
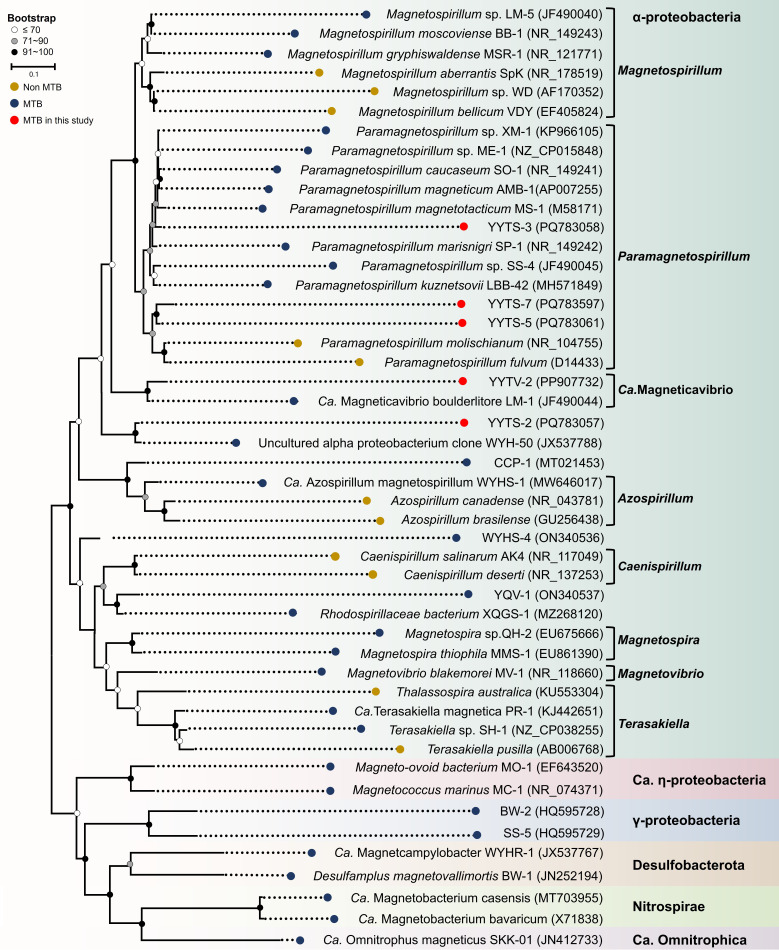
Maximum-likelihood phylogenetic tree based on 16S rRNA gene sequences, highlighting the MTB detected in Yuyuantan Lake sediments. Representative families within *Alphaproteobacteria* (e.g., *Magnetospirillaceae*, *Azospirillaceae*, *Rhodospirillaceae*, *Magnetospiraceae*, *Magnetovibrionaceae*, and *Terasakiellaceae*) are labeled for taxonomic context, with all analyzed MTB strains falling within the *Rhodospirillales* order of this class. Brackets on the right indicate genera, and background colors denote distinct phyla. Bootstrap support values (from 1,000 replicates) are indicated at the nodes. GenBank accession numbers are shown in parentheses. The scale bar corresponds to 10% sequence divergence. MTB strains from other phyla are used as the outgroup to root the tree.

Phylogenetic placement based on 16S rRNA (Fig. 1) assigned all five lineages to *Magnetospirillaceae*. Strains YYTS-3, YYTS-5, and YYTS-7 clustered within *Paramagnetospirillum*, whereas YYTS-2 grouped with clone WYH-50, supporting its status as a candidate novel genus. For YYTV-2, whole-genome metrics substantiated species-level novelty within *Ca*. Magneticavibrio: the ANI to *Ca*. Magneticavibrio boulderlitore LM-1 was 90.52% (EzBioCloud), and the AAI was 92.44% (Enveomics Gateway), both below species-level thresholds (95% for ANI and approximately 95–96% for AAI), and consistent with its 16S rRNA gene similarity (96.61%). Draft genomes for YYTS-2, YYTS-3, YYTS-5, and YYTS-7 were not recovered, likely due to limited input DNA.

To resolve the morphologies of the five MTB lineages, we designed species-specific oligonucleotide probes and performed coupled FISH-SEM experiments (Table S2). Fluorescence microscopy confirmed probe specificity: (i) rod-shaped *E. coli* cells hybridized with the universal bacterial EUB338 probe (green) in all assays, (ii) vibrio cells (strain YYTV-2) hybridized with both EUB338 (green) and the species-specific YYTV2-920 probe (red), identifying a vibrio-type MTB (Fig. S2a and b), and (iii) spiral cells (strains YYTS-2, YYTS-3, YYTS-5, and YYTS-7) each hybridized with their respective species-specific probes (YYTS2-1106, YYTS3-924, YYTS5-917, and YYTS7-532), supporting species-level assignments (Fig. S2e and f, i and j, m and n, q and r).

SEM imaging corroborated a distinct vibrioid morphology for YYTV-2 (from microcosm 1; Fig. S2c and d). In contrast, YYTS-2, YYTS-3, YYTS-5, and YYTS-7 were indistinguishable by morphology alone because all were spirilla bearing a single magnetosome chain with similar crystal shapes (Fig. S2g and h, k and l, o and p, s and t). Also, these strains co-occur within the microcosms (YYTS-2/YYTS-3 in microcosm 1; YYTS-5/YYTS-7 in microcosm 2; Table S1). Thus, while FISH-SEM reliably verifies strain identity, the morphological similarity among spirilla limits discrimination; finer classification, therefore, requires complementary molecular, TEM, and genomic analyses.

### Morphological and genomic characterization of strain YYTV-2

**Fig 2:**
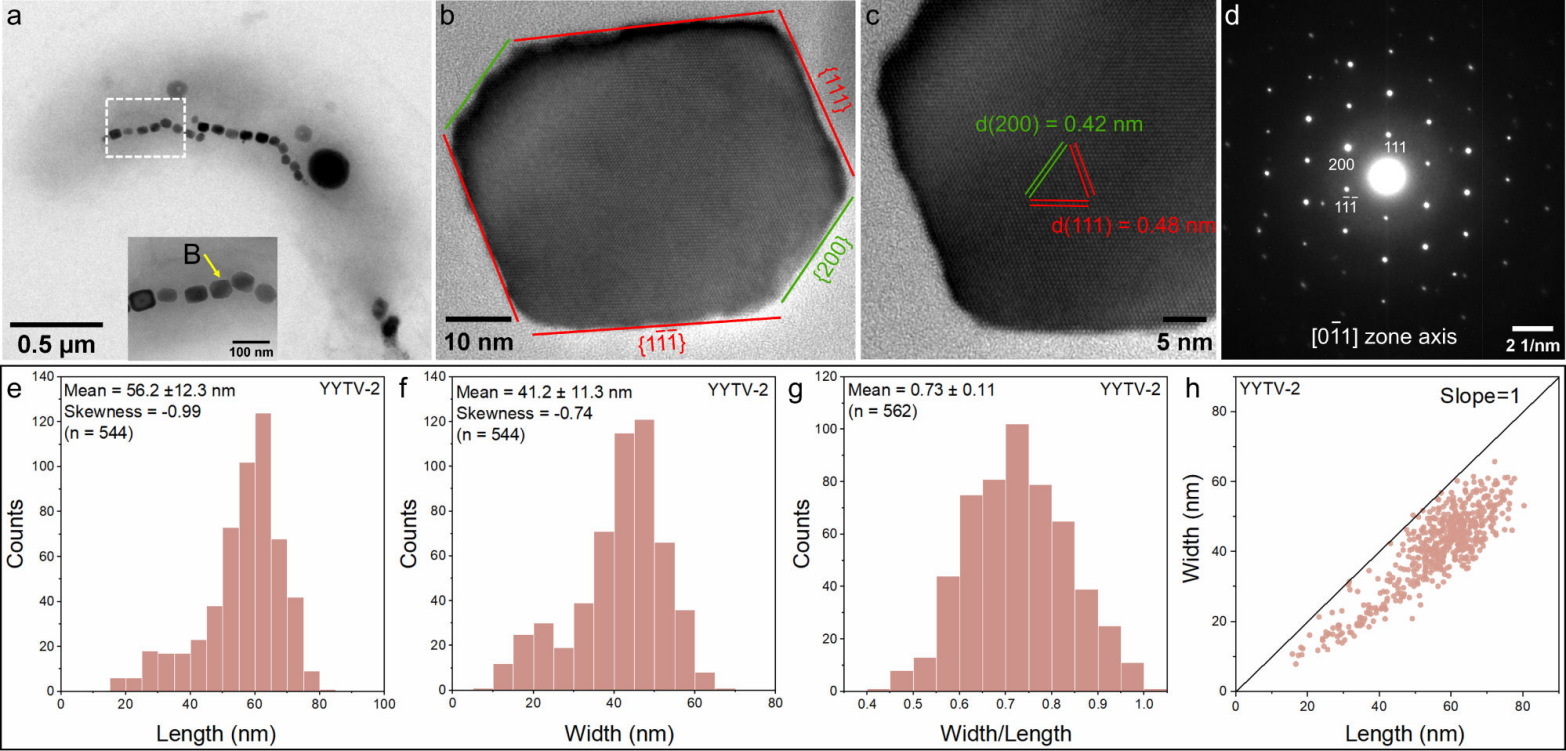
Morphological and structural characterization of strain YYTV-2. (**a**) TEM image of a YYTV-2 cell, with (inset) zoom-in on the magnetosome chain (in the white dashed box). (**b**) HRTEM image of a magnetosome particle (indicated by yellow arrow in the (**a**) inset), recorded along the $0\bar{1}1$ zone axis, with crystal facets and Miller indices labeled. (**c**) HRTEM image of the lower left region from (**b**)), with corresponding lattice spacings. (**d**) SAED pattern for the magnetosome in (**b**) with Miller indices. (**e**) Histogram of magnetosome crystal lengths in YYTV-2. (**f**) Histogram of magnetosome crystal widths. (**g**) Histogram of shape factors (width/length) for YYTV-2 magnetosomes. (**h**) Scatter plot of crystal length versus width for YYTV-2 magnetosomes, with particle size distribution.

**Fig 3:**
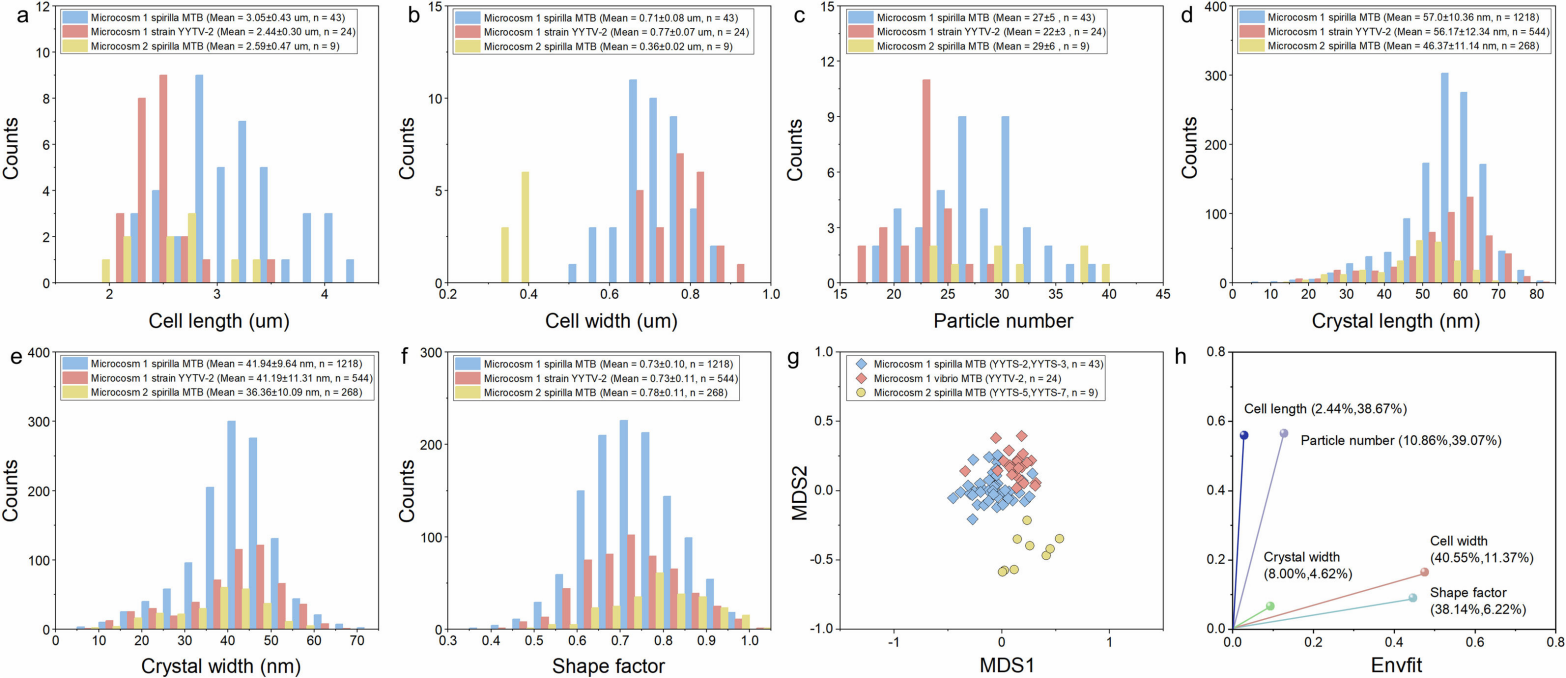
Micromorphological features of MTB strains from microcosms 1 and 2. (**a–f**) Histograms of the morphological characteristics of spirilla cells (43 individual TEM cells), vibrio cells (24 TEM cells of strain YYTV-2) from microcosm 1, and spirilla cells from microcosm 2 (9 TEM cells). (**a**) Cell length, (**b**) width, (**c**) particle number for spirilla and vibrio cells in both microcosms. (**d**) Crystal length, (**e**) width, and (**f**) shape factor (width/length ratio) of magnetosomes in the same strains. Spirilla cells from (blue) and vibrio cells (pink; strain YYTV-2) from microcosm 1, and spirilla cells from microcosm 2 (yellow) are indicated. (**g**) Non-metric multidimensional scaling (nMDS) ordination plot based on cell and magnetosome morphological features (cell length, cell width, particle number, crystal width, and shape factor) for MTB from microcosms 1 (67 TEM cells analyzed) and 2 (9 TEM cells analyzed). Euclidean distance was used, with a stress value of 0.15. Spirilla cells from microcosm 1 (43 TEM cells) and strain YYTV-2 (24 TEM cells) are represented by blue and pink diamonds, respectively; spirilla cells (9 TEM cells) from microcosm 2 are represented by yellow circles. (**h**) Environmental fitting (Envfit) analysis based on nMDS results. Each color represents a specific morphological feature. Percentages in parentheses next to each feature indicate the proportion of variation attributed to the feature along nMDS1 and nMDS2, respectively.

TEM showed that YYTV-2 produces magnetite-type magnetosomes with a mean length of 56.2 ± 12.3 nm, a mean width of 41.2 ± 11.3 nm, and a shape factor (width/length) of 0.73 ± 0.11 (*n* = 544 magnetosomes from 24 cells) (Fig. 2e through h). HRTEM and SAED identified Fe_3_O_4_ and a slightly elongated cubo-octahedral habit, consistent with the measured shape factor (Fig. 2a through d). In vibrioid cells, magnetosomes form a single chain along the positive curvature, with 22 ± 3 crystals per cell (*n* = 24). Host cells measured 2.44 ± 0.3 µm in length and 0.77 ± 0.07 µm in width (*n* = 24) (Fig. 3a through c).

**Fig 4:**
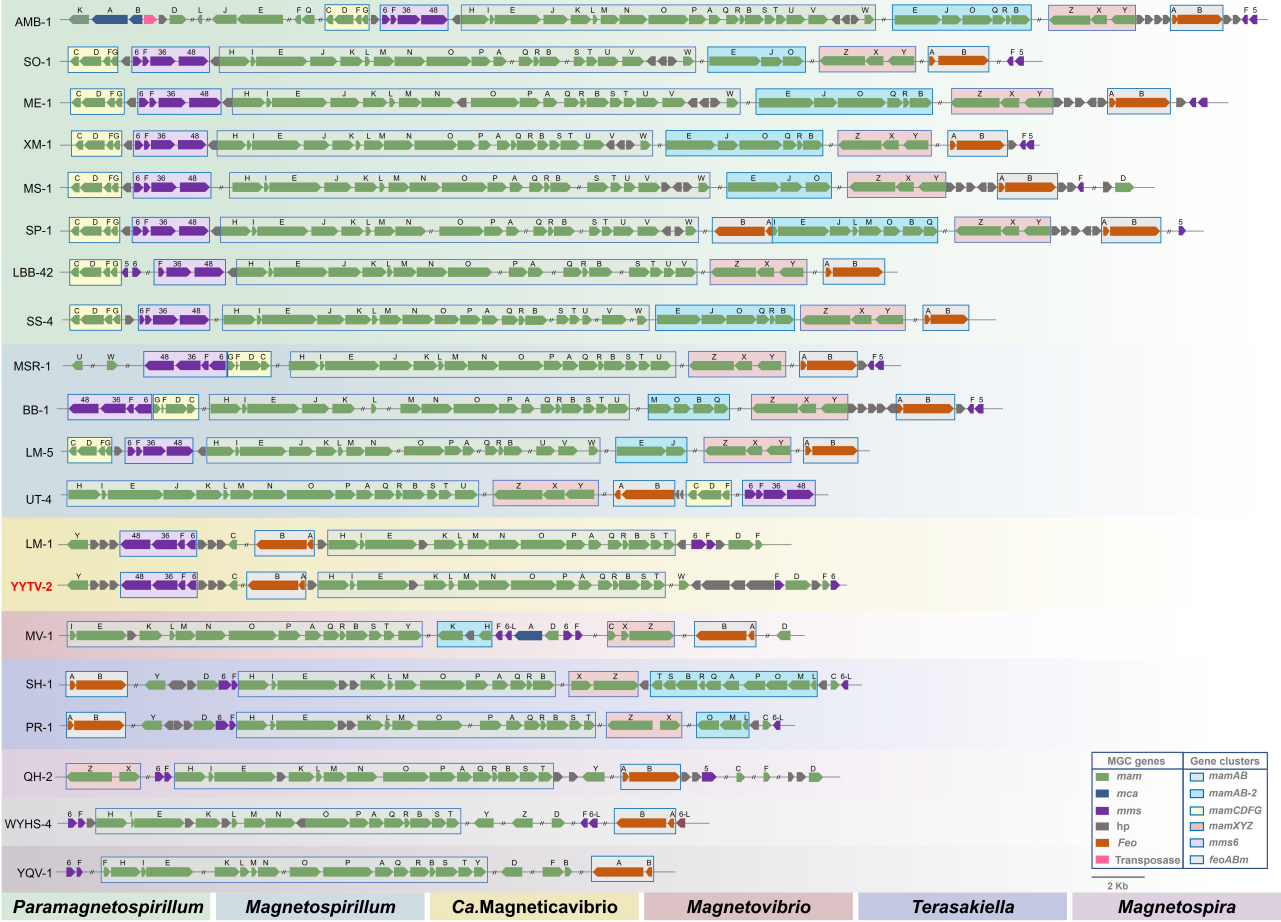
Gene organization of MGCs in representative magnetotactic *Alphaproteobacteria* strains. Strains from the same genus have the same background color. WYHS-4 and YQV-1 represent two newly proposed unnamed *Alphaproteobacteria* genera.

A high-quality draft genome was recovered for YYTV-2 (∼98.5% completeness, negligible contamination) from metagenomic sequencing of magnetically isolated bacteria (microcosm 1), followed by binning and reassembly. This assembly totals ~3.4 Mb (66 contigs; GC content 60.35%). BLAST annotation revealed a complete complement of magnetosome-related genes (e.g., *mam*, *mms*, and *feo*) arranged mainly in five clusters: *mamAB*, *mamXYZ*, *mamCDFG*, *mms6*, and *feoABm* (Fig. 4). The *mamAB* region comprises *mamH*, *mamI*, *mamE*, *mamK*, *mamL*, *mamM*, *mamN*, *mamO*, *mamP*, *mamA*, *mamQ*, *mamR*, *mamB*, *mamS*, and *mamT*. The *feoABm* cluster (*feoA* and *feoB*) lies upstream of *mamAB*, separated by a hypothetical gene. Overall gene content and synteny in *mamAB* and *feoABm* resemble those of other magnetotactic *Alphaproteobacteria* (Fig. 4).

### Morphological characteristics of the other four MTB strains

We compared YYTV-2 with four spirilla (YYTS-2, YYTS-3, YYTS-5, and YYTS-7). Coupled FISH-SEM confirmed strain-level identities, but the spirilla were indistinguishable by SEM alone because all exhibited a single magnetosome chain with similar crystal habits and co-occurred within samples (YYTS-2/YYTS-3 in microcosm 1; YYTS-5/YYTS-7 in microcosm 2). To resolve fine-scale differences, we analyzed 52 spirilla by TEM (43 from microcosm 1; 9 from microcosm 2) (Fig. S3 and S4, and Table S7).

Spirilla from microcosm 1 had cell lengths of ~2.2–4.3 µm (Fig. 3a) and contained 16–39 magnetosomes per cell (Fig. 3c); crystal dimensions were 53–61 nm (length), 38–44 nm (width), and 0.68–0.83 (width/length) (Fig. 3d through f). By comparison, spirilla from microcosm 2 (YYTS-5/YYTS-7) measured ~2.1–3.8 µm with 16–38 magnetosomes per cell; crystals were 40–57 nm (length), 31–45 nm (width), and 0.76–0.81 (width/length) (Fig. 3a, c through f). HRTEM showed elongated cubo-octahedral crystals in all spirilla, similar to YYTV-2 (Fig. S5).

To differentiate vibrio YYTV-2 from spirilla and to compare microcosms, we performed nMDS on five TEM-derived traits (cell length, cell width, particle number, crystal width, and shape factor). Environmental fitting (Envfit) analysis indicated that cell length and magnetosome number were most associated with the MDS2 axis, accounting for 38.7% and 39.1% of variation, respectively. In contrast, magnetosome crystal length, width, and width/length primarily loaded on the MDS1 axis, with contributions of 4.6%, 11.4%, and 6.2%, respectively (Fig. 3h). nMDS separated spirilla from microcosm 2 from YYTV-2 plus spirilla from microcosm 1 (Fig. 3g). Thus, the five strains cannot be reliably distinguished by magnetosome morphology alone; microcosm-level shifts likely reflect modest environmental differences affecting growth and biomineralization (59).

### Correlation between MTB phylogeny and biomineralization

**Fig 5:**
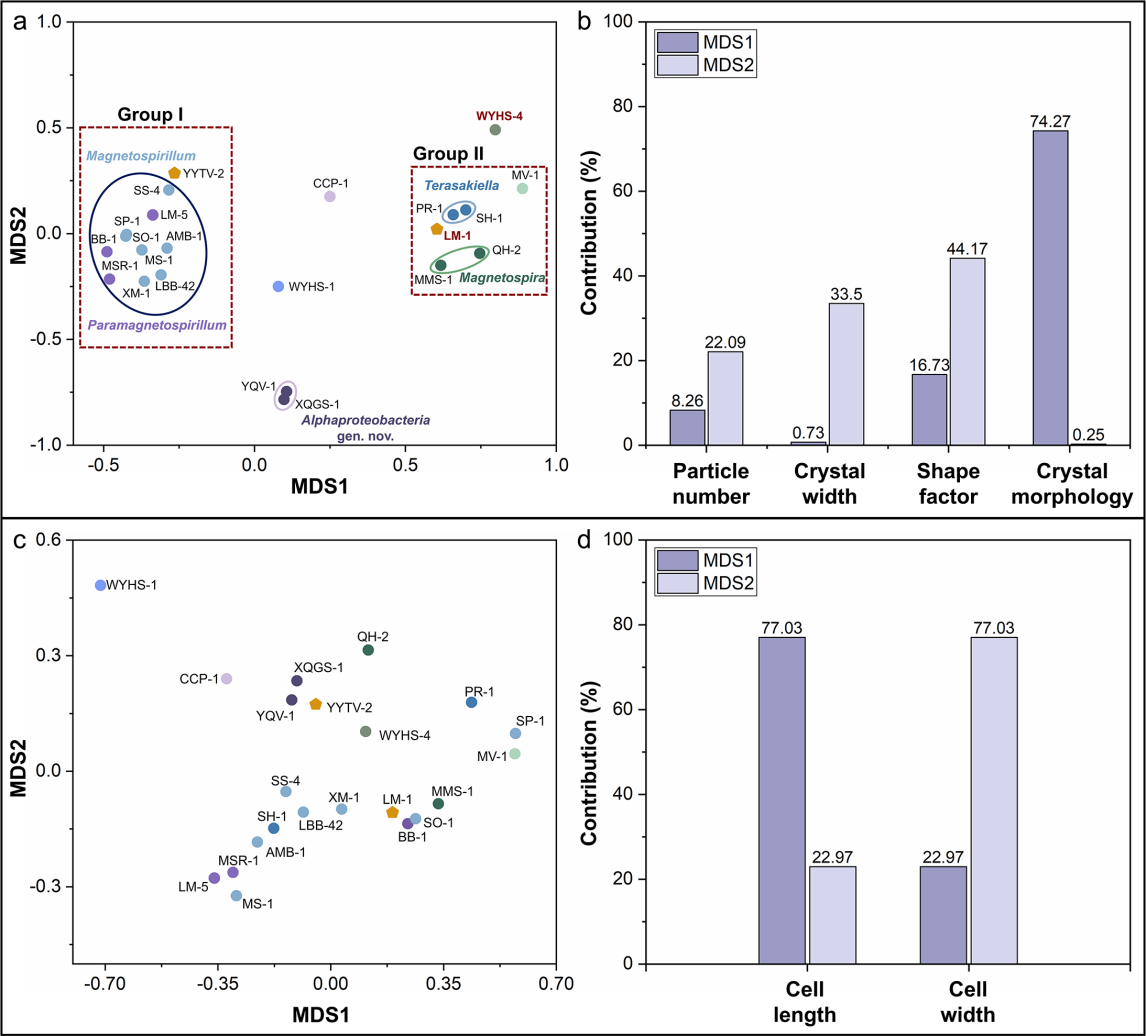
nMDS analyses of magnetosome and cell morphological features in *Alphaproteobacteria* MTB. (**a**) nMDS plot of four morphological features (crystal number, morphology, width, and shape factor) based on Euclidean distance (stress value = 0.096). Red dashed rectangles mark phylogenetic Groups I and II, which are defined by the topology of Fig. 1 combined with clustering tendencies in the nMDS analysis. Strains WYHS-4 and LM-1, which cluster with Group II despite not belonging to this group based on phylogenetic topology, are highlighted in bold red. Solid-line ovals denote genera: *Paramagnetospirillum* and *Magnetospirillum* (dark blue), *Magnetospira* (green), *Terasakiella* (light blue), and the unnamed genus containing XQGS-1 and YQV-1 (purple). See Table S8 for the full strain list, references, and TEM data. Note that proximity in the nMDS plot reflects magnetosome trait similarity rather than taxonomic relatedness. (**b**) Magnetosome trait contributions to nMDS axes based on environmental fitting (Envfit) analysis. Dark purple bars are contributions to MDS1, light purple bars to MDS2. (**c**) nMDS plot of cell morphology (cell length and width) based on Euclidean distance (stress = 0, consistent with two-dimensional input). Strains are color-coded by genus, consistent with (**a**). (**d**) Contributions of cell morphological features to the nMDS axes based on Envfit analysis. Dark purple bars are contributions to MDS1, light purple bars to MDS2. Cell length contributes mainly to MDS1 (~77.0%), whereas cell width contributes mainly to MDS2 (~77.0%).

*Alphaproteobacteria* MTB resolved into two primary groups (Groups I and II) based on 16S rRNA phylogenetic tree topology (Fig. 1), broadly consistent with genome-based phylogeny (Fig. S6a) and with clustering patterns in nMDS of magnetosome trait (Fig. 5a). Group I includes *Magnetospirillum* (MSR-1, LM-5, and BB-1), *Paramagnetospirillum* (AMB-1, XM-1, MS-1, SO-1, LBB-42, SP-1, and SS-4) and *Ca*. Magneticavibrio (YYTV-2 and LM-1) genera (27, 60–63). Group II encompasses *Terasakiella* (PR-1 and SH-1) and *Magnetospira* (QH-2 and MMS-1), along with the *Magnetovibrio* strain MV-1 (25, 34, 64–66). Several strains XQGS-1, YQV-1, WYHS-1, CCP-1, and WYHS-4 do not align with either phylogenetic group (12, 21, 26, 67) (Fig. 5a).

Separate nMDS analyses of magnetosome traits versus cell morphology underscored stronger phylogenetic signal in biomineralization features (Fig. 5; Table S8). For magnetosome traits (particle number, crystal width, shape factor, and crystal morphology), Groups I and II were distinctly separated along MDS1; unclassified strains (XQGS-1, YQV-1, WYHS-1, and CCP-1) plotted between groups (Fig. 5a). Within groups, congeners or closely related genera clustered (e.g., *Magnetospirillum* with *Paramagnetospirillum* in Group I; *Magnetospira* with *Terasakiella* in Group II), as did YQV-1 and XQGS-1 from an unidentified genus (67, 68). In contrast, nMDS based on cell size (length and width) showed weaker separation and less consistent genus-level clustering (Fig. 5c). Overall, these results indicate that magnetosome features carry a stronger phylogenetic signal than cell morphology, consistent with previous observations that biomineralization traits are more evolutionarily conserved in MTB (8, 19, 68).

Envfit identified crystal habit as the dominant driver of the MDS1 separation (variance explained ~74.3%), with crystal width loading most strongly on MDS2 (~44.2%) (Fig. 5b). For cell morphology, cell length predominated on the MDS1 axis (~77.0%), with cell width on the MDS2 axis (~77.0%) (Fig. 5d). Thus, crystal habit (e.g., elongated cubo-octahedral vs prismatic) differentiates major phylogenetic groups, whereas size-related traits (width and particle number) largely drive within-group variation.

Notably, WYHS-4 and LM-1 cluster with Group II in the nMDS of magnetosome trait despite limited phylogenetic affinity, reflecting convergence on prismatic magnetite morphologies (34, 64–67, 69). This emphasizes that nMDS clustering reflects similarity in magnetosome traits rather than taxonomic relatedness (Fig. 5a). Across Groups I and II, TEM/HRTEM shows broadly similar crystal sizes (~30–58 nm; Table S8), and MDS2 does not resolve taxa by size alone (Fig. 5a). In Group I, *Magnetospirillum*, *Paramagnetospirillum*, and YYTV-2 predominantly form cubo-octahedral to elongated cubo-octahedral magnetite magnetosomes (27, 60–63, 70, 71), whereas strains WYHS-1, CCP-1, YQV-1, and XQGS-1 yield truncated cubo-octahedral crystals (27, 67, 68). Taken together, the nMDS and Envfit results quantitatively support that magnetosome traits carry stronger phylogenetic signal than cell morphology in the updated *Alphaproteobacteria* taxonomy, while also revealing morphological convergence driven by crystal habit (27, 67, 68).

### Whole-genome versus magnetosome-operon phylogenies at the genus level

We compare phylogenies inferred from whole genomes and from magnetosome operons (*mamAB* and *mamAB-2*) (Fig. S6). At the genus level, the topologies are broadly congruent: *Magnetospira*, *Ca*. Magneticavibrio, *Terasakiella*, and *Magnetovibrio* form monophyletic clades in both trees. Notable exceptions indicate locus-specific histories. Strains WYHS-4 and QH-2 shift positions between the genome-based and operon-based trees, and cross-genus incongruence is evident for LM-5, SP-1, and MS-1 within *Magnetospirillum*/*Paramagnetospirillum*: LM-5 clusters with *Magnetospirillum* in the genome tree but affiliates with *Paramagnetospirillum* in the operon tree, whereas SP-1 and MS-1 show the opposite pattern. Taken together, these results support predominantly vertical inheritance of magnetosome loci at the genus scale, punctuated by occasional cross-genus replacements or transfer of *mamAB* and *mamAB-2*.

## DISCUSSION

### Genetic basis for magnetosome formation in *Alphaproteobacteria* MTB

Current models partition magnetosome biomineralization into discrete steps controlled by gene subsets: *mamBILQ* (vesicle formation), *feoAB* and *mamBMHZ* (iron import), *mamOPTSZ* (magnetite deposition/redox control), and *mamKY* (chain organization) (29, 34). These modules are conserved across *Alphaproteobacteria* MTB and likely underwrite the recurrent phenotype of a single magnetosome chain.

Genes implicated in crystal habit include *mms6* and *mamD*, which are widely conserved, yet crystal shape and size still track phylogeny. In our data set, most Group I taxa (except LM-1) form cubo-octahedral magnetite, whereas Group II taxa, and WYHS-4 and LM-1, form prismatic crystals. Notably, YYTV-2 and LM-1 (both *Ca*. Magneticavibrio) share similar MGC content and synteny, but YYTV-2 produces elongated cubo-octahedra while LM-1 forms prisms (25). This decoupling of gene content from habit points to regulatory layers beyond core MGCs. Candidates include Mms6 (promotes well-ordered cubo-octahedra) and cofactors such as MamGFDC or MmsF (which can bias elongation toward prismatic morphologies) (28, 72). Environmental variables (e.g., O_2_ and Fe availability) likely modulate these genetic programs (59). These hypotheses are testable once genetic tools become available for prismatic-forming MTB, including YYTV-2.

A core *mamCFPRST* module is conserved across magnetotactic *Alphaproteobacteria* MTB and probably underlies baseline size regulation. Accessory genes *mms36* and *mms48*, largely restricted to *Magnetospirillum*, *Paramagnetospirillum*, and *Ca*. Magneticavibrio (18, 20, 29, 67), correlate with narrower crystal sizes (≈29.7–53.3 nm) relative to broader ranges in lineages lacking these genes (≈32.8–83.3 nm) (17, 29, 34). While correlative, this pattern suggests that *mms36*/*mms48* constrain crystal size variation; causal roles require experimental validation.

A duplicated *mamAB-2* cluster, present in many *Paramagnetospirillum* and other alphaproteobacterial MTB, has been linked to increased crystal size and particle number (34), consistent with experimental amplification of *mamAB* in *M. gryphiswaldense* (73). The origin of *mamAB-2* via duplication or HGT (74) provides a mechanism for within-genus heterogeneity in magnetosome traits and helps explain the genus-level variability in particle number and size captured by our nMDS analyses (Fig. 5a). Comparisons of whole-genome and operon (*mamAB*/*mamAB-2*) phylogenies indicate broad genus-level congruence, consistent with predominant vertical inheritance, but with locus-specific incongruence in several strains (e.g., LM-5, SP-1, MS-1, WYHS-4, and QH-2). These patterns are most parsimoniously explained by occasional cross-genus operon replacement or transfer and by gene duplication, superimposed on a vertically inherited framework.

Conserved MGC architectures establish a common scaffold for vesicle formation, iron trafficking, redox control, and chain organization, yielding broadly similar chain configurations across related MTB. Superimposed on this scaffold, duplication/HGT of magnetosome loci and environmental modulation introduce quantitative variation in crystal size and number and qualitative shifts in crystal habit. Consistent with earlier observations (8, 19, 68), our quantitative analyses show that magnetosome traits carry a stronger phylogenetic signal than cell morphology, indicating that genetic conservation and genomic innovation jointly shape biomineralization diversity in alphaproteobacterial MTB. These results generate testable predictions: (i) perturbing regulatory factors (e.g., Mms6/MmsF/MamGFDC) should shift habit within a conserved MGC background; (ii) experimental duplication of *mamAB*-like loci should increase particle number/size in multiple lineages; and (iii) controlled O_2_/Fe manipulations should modulate trait envelopes without altering core synteny.

### Evolution of magnetosome operons in the *Magnetospirillaceae*

**Fig 6:**
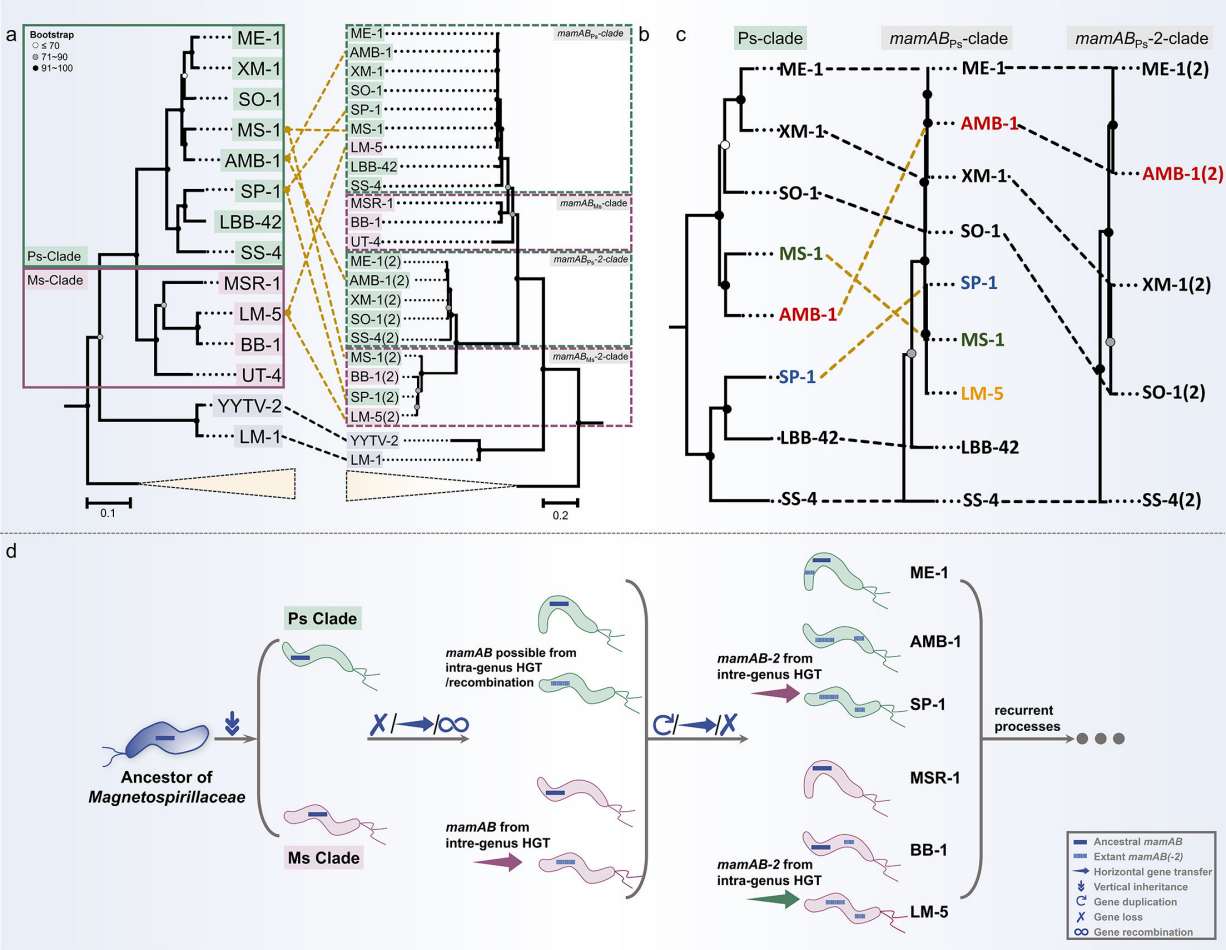
Evolutionary scenarios of magnetosome operons in the family *Magnetospirillaceae*. (**a and b**) Topological comparisons of *Magnetospirillaceae* and *Ca*. Magneticavibrio MTB phylogenetic trees based on (**a**) whole-genome and (**b**) magnetosome core gene sequences, including both the *mamAB* operon and *mamAB-2* cluster. Both trees were constructed using the maximum likelihood method, with *Gammaproteobacteria* MTB strains as outgroups. Bootstrap values (1,000 replicates) are shown at nodes. Scale bars indicate 10% in (**a**) and 20% in (**b**), respectively. For the *mamAB-2* phylogeny, a conserved subset of genes (*mamE*, *mamJ*, *mamO*, *mamQ*, *mamR*, and *mamB*) present in ≥45% of alphaproteobacterial MTB was used. (**b**) Sequence divergence. (**C**) Enlarged views of the *Paramagnetospirillum* (PS), *mamAB*_Ps_, and *mamAB*-2_Ps_ clades from (**a and b**). (**d**) Conceptual evolutionary model of magnetosome operons in *Magnetospirillaceae*. Green arrows denote intra-genus HGT events, and purple arrows inter-genus HGT events. Gray arrows trace the main evolutionary trajectories from the ancestral lineage toward descendant lineages. Blue symbols along these trajectories indicate evolutionary processes that can arise repeatedly during diversification (vertical inheritance, gene loss, gene recombination, horizontal gene transfer, and duplication). The ellipsis on the right margin represents the potential for these processes to recur, driving further diversification of magnetosome operons.

At the genus level, the comparisons of genome versus operon-based phylogenies (Fig. S6) show broad congruence across *Alphaproteobacteria*, consistent with predominantly vertical inheritance of the canonical *mamAB* operon (18, 19, 22, 31, 75). Within *Magnetospirillaceae*, however, several strains deviate from this pattern, revealing striking locus-specific incongruences. To resolve these discrepancies, we analyzed both the canonical *mamAB* and its duplicated counterpart *mamAB-2* (Fig. 6).

In the genome tree, *Magnetospirillaceae* form two monophyletic clades corresponding to the genera *Magnetospirillum* (hereafter Ms) and *Paramagnetospirillum* (hereafter Ps) (Fig. 6a). In contrast, operon phylogenies resolve four clades (Fig. 6b): *mamAB*_Ps_ and *mamAB*_Ms_ for the canonical core operon, and *mamAB*-2_Ps_, and *mamAB*-2_Ms_ for duplicated or horizontally acquired homologs. Most strains retain concordant positions, but several show clear mismatches. For instance, LM-5 clusters with Ms in the genome tree, while its *mamAB* groups with *mamAB*_Ps_ (Fig. 6a and b). Similarly, AMB-1 groups with MS-1 in the genome tree, yet its *mamAB* operon clusters with ME-1 in the *mamAB*_Ps_ clade. These incongruences indicate that *mamAB* evolution cannot be explained by vertical inheritance alone and likely involves HGT and/or recombination (27).

Comparisons between *mamAB* and *mamAB-2* reveal further complexity. In most lineages, *mamAB*_Ps_ and *mamAB-2*_Ps_ (or *mamAB*_Ms_ and *mamAB-2*_Ms_) tend to be congruent, consistent with vertical transmission. Exceptions include SP-1, whose *mamAB-2* resides within *mamAB-2*_Ms_ rather than *mamAB-2*_Ps_ (Fig. 6b and c), implying inter-genus replacement. MS-1, assigned to the Ps in the genome tree, also harbors *mamAB-2* within the *mamAB-2*_Ms_. LM-5 is particularly illustrative: although genomic data place it in Ms, its *mamAB* groups with *mamAB*_Ps_, whereas *mamAB-2* affiliates with *mamAB-2*_Ms_. This pattern suggests that LM-5 replaced its ancestral *mamAB* with a Ps-type *mamAB* through inter-genus HGT, while retaining or subsequently reacquiring an Ms-type *mamAB-2* through intra-genus transfer. UT-4 persistently branches basally in operon trees (Fig. 6a and b), in line with proposals that it preserves an ancestral configuration (27, 31).

Synthesizing these patterns (Fig. 6d), we infer that the *Magnetospirillaceae* ancestor carried a single *mamAB* operon inherited vertically into Ps and Ms after their split. Subsequent evolution was non-linear: some lineages retained the ancestral operon (e.g., ME-1 and MSR-1), whereas others underwent gene loss and reacquisition of *mamAB* through HGT or recombination (e.g., AMB-1 and SP-1). The canonical *mamAB* subsequently gave rise to *mamAB-*2 via duplication (e.g., ME-1 and BB-1) and by replacement through intra- or inter-genus transfer (e.g., LM-5). This framework accords with the view that operon duplication and HGT are recurrent forces shaping MGCs (17, 34) and underscores the frequent interplay among vertical inheritance, gene loss, and operon replacement.

Collectively, our results emphasize that magnetosome operon diversification in *Magnetospirillaceae* reflects a dynamic balance between conservation and innovation. Vertical inheritance preserved the essential genetic basis of magnetotaxis, while recurrent duplication and horizontal replacement introduced lineage-specific variability in biomineralization. This duality reconciles the conservation of core magnetosome functions with the notable structural flexibility of magnetosome operons across closely related genera.

### Conclusions

We characterized five uncultured *Rhodospirillales* MTB and reconstructed a high-quality genome for vibrioid strain YYTV-2. 16S rRNA phylogeny places four strains within or near *Paramagnetospirillum*, expanding diversity in this lineage, whereas YYTV-2 represents a novel species of the rarely studied *Ca*. Magneticavibrio genus. Despite this shared genus, YYTV-2 and LM-1 differ in magnetosome crystal habit (elongated cubo-octahedral vs prismatic), a divergence not previously reported for *Alphaproteobacteria* MTB. Comparative analyses of MGCs indicate that variation in accessory modules outside the core *mamAB* operon, together with the presence/absence and type of *mamAB-2*, likely contributes to these morphological differences. By jointly analyzing both the canonical *mamAB* and duplicated *mamAB-2* operons, our comparative framework reveals incongruences between genome-based and operon-based phylogenies. The patterns support a model in which vertical inheritance dominates at the genus level, but recurrent operon duplication and horizontal exchange, including inter-genus replacement, have remodeled MGCs in specific lineages. Thus, conserved genetic architectures maintain the core magnetotaxis program, while genomic plasticity introduces lineage-specific diversification in crystal habit, particle number, and size. These results refine evolutionary models of magnetosome operons and strengthen the view that magnetosome traits carry a stronger phylogenetic signal than cell morphology, improving the interpretive power of MTB and magnetofossils in ecological and evolutionary studies.

## Data Availability

The 16S rRNA gene sequence and the genome of strain YYTV-2 have been assigned to GenBank accession numbers PP907732 and JBHMJB000000000, respectively. The GenBank accession numbers for the 16S rRNA gene sequences of strains YYTS-2, YYTS-3, YYTS-5, and YYTS-7 are PQ783057, PQ783058, PQ783061, and PQ783597, respectively.

## SUPPLEMENTAL MATERIAL

Supplemental material aem.02121-25-s0001.pdf Figures S1 to S6; Tables S1 to S8.

ASM does not own the copyrights to Supplemental Material that may be linked to, or accessed through, an article. The authors have granted ASM a non-exclusive, world-wide license to publish the Supplemental Material files. Please contact the corresponding author directly for reuse.
