# Supplementary material for: Phylogenetic and genomic insights into magnetosome biomineralization in magnetotactic Alphaproteobacteria
Source: Appl Environ Microbiol. 2025 Nov 28;91(12):e02121-25. doi: 10.1128/aem.02121-25 (PMC12724286; doi:10.1128/aem.02121-25)
Supplement: Supplemental material — Figures S1 to S6; Tables S1 to S8. [file aem.02121-25-s0001.pdf]

Supplementary materials

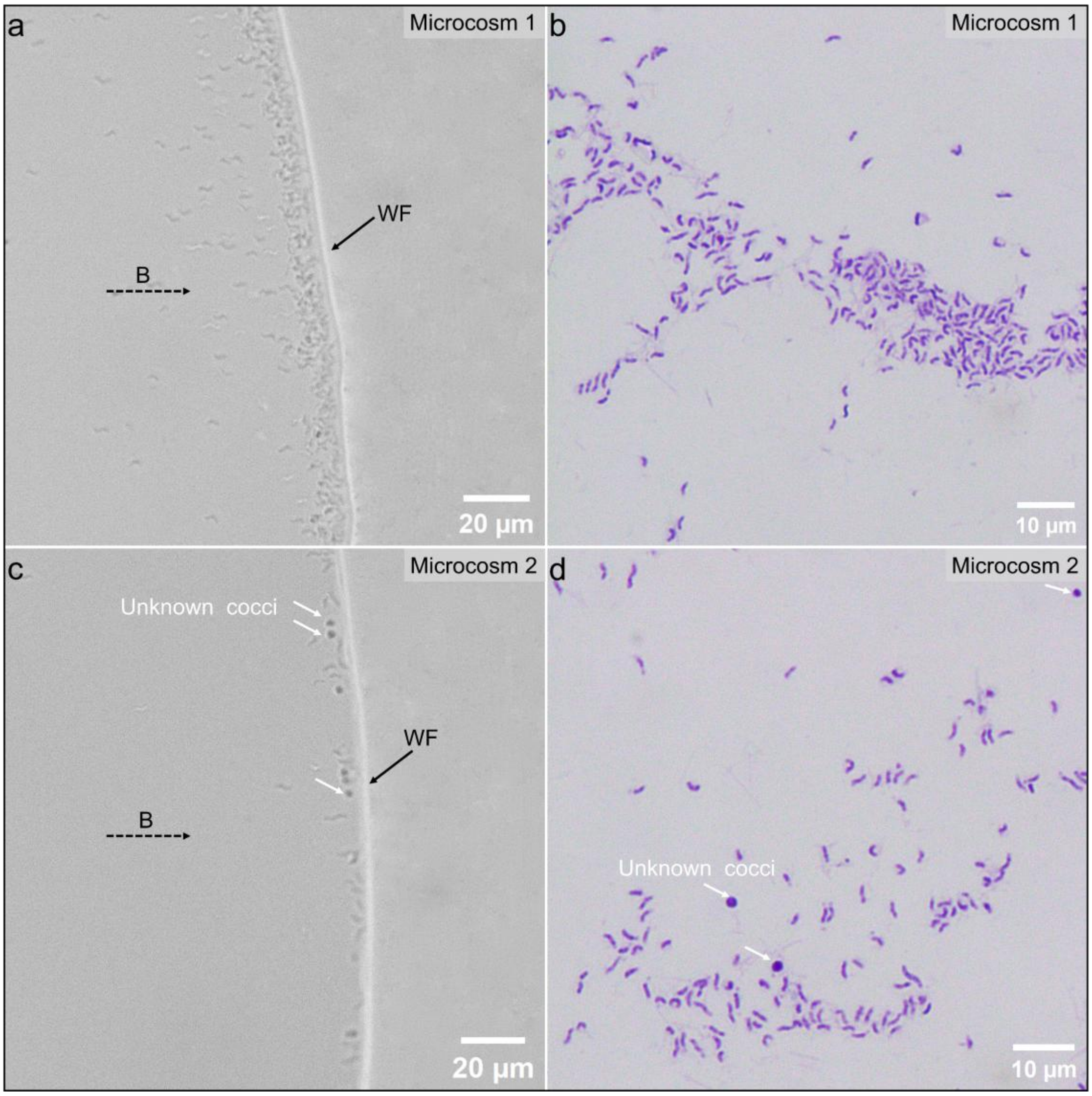

Fig. S1 Morphological diversity of MTB in Lake Yuyuantan sediments. (a) and (c) Optical microscope image of living MTB cells swimming out from a small sediment drop on the left (sediment not shown) along magnetic field lines (dashed line with arrow) and gathering at the water droplet edge (water front, WF) in microcosms 1 and 2, respectively. (b) and (d) Optical microscopy images of crystal violet-stained MTB cells magnetically separated from microcosms 1 and 2, respectively. Cocci cells in microcosm 2 are indicated by white arrows. The spirillum and vibrio MTB cells make up ~98.50% and ~97.85% of the MTB population in microcosm 1 (n = 133) and 2 (n = 93), respectively.

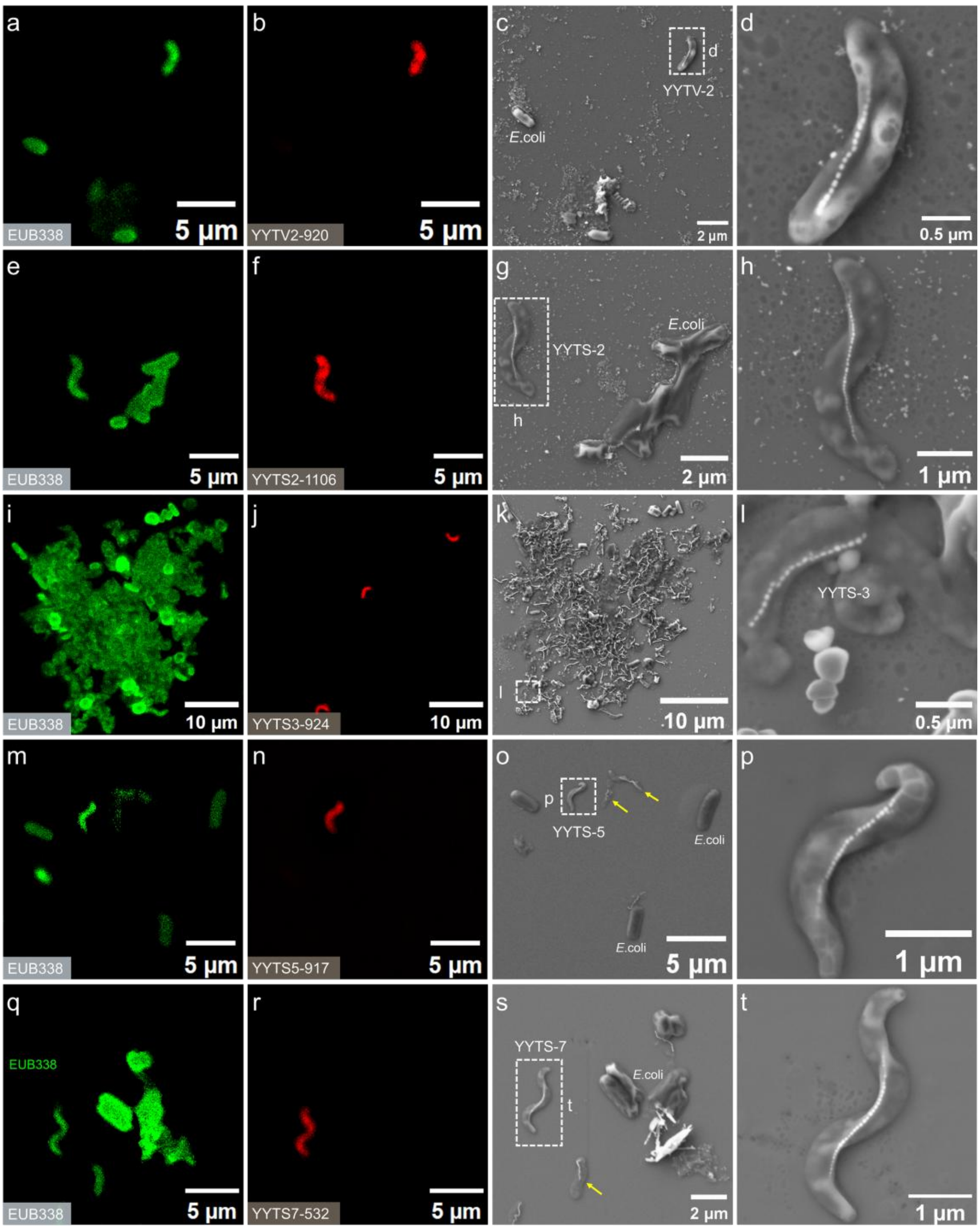

Fig. S2 Phylogenetic and structural identification of magnetotactic vibrio and spirilla cells from microcosms 1 and 2. (a–d) YYTV-2, (e–h) YYTS-2, (i–l) YYTS-3, (m–p) YYTS-5, and (q–t) YYTS-7 were analyzed using the correlative FISH-SEM approach. First column: fluorescence microscopy images of bacteria hybridized *in situ* with the 5′-FAM-labelled bacterial universal probe EUB338. Second column: fluorescence micrographs of bacterial cells hybridized *in situ* with the 5′-Cy3-labelled species-specific probes. Third column: SEM images of the same areas as in the first column. Fourth column: High-magnification SEM image of the targeted magnetotactic spirilla, highlighted by dashed boxes in the third column. Strains YYTV-2, YTTS-2, and YTTS-3 are from microcosm 1, and strains YTTS-5 and YTTS-7 are from microcosm 2. Cells indicated by yellow arrows in microcosm 2 (Fig. S2o and S2s) with small size and ambiguous morphology are an unidentified strain.

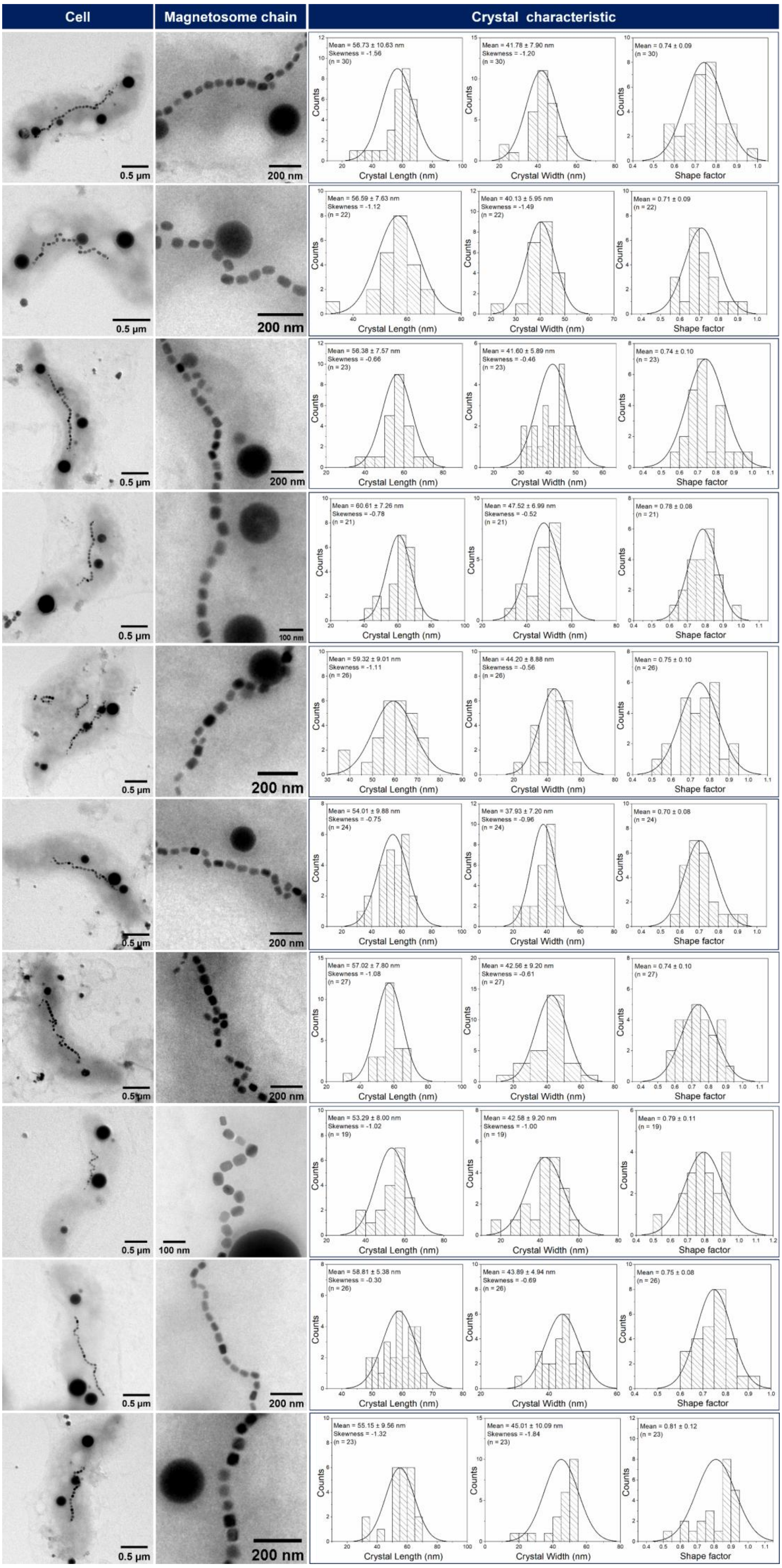
Cell
Magnetosome chain
Crystal characteristic
0.5 µm
200 nm
100 nm
Counts
Crystal Length (nm)
Crystal Width (nm)
Shape factor
Mean = 56.73 ± 10.63 nm
Skewness = -1.56
(n = 30)
Mean = 41.78 ± 7.90 nm
Skewness = -1.20
(n = 30)
Mean = 0.74 ± 0.09
(n = 30)
Mean = 56.59 ± 7.63 nm
Skewness = -1.12
(n = 22)
Mean = 40.13 ± 5.95 nm
Skewness = -1.49
(n = 22)
Mean = 0.71 ± 0.09
(n = 22)
Mean = 56.38 ± 7.57 nm
Skewness = -0.66
(n = 23)
Mean = 41.60 ± 5.89 nm
Skewness = -0.46
(n = 23)
Mean = 0.74 ± 0.10
(n = 23)
Mean = 60.61 ± 7.26 nm
Skewness = -0.78
(n = 21)
Mean = 47.52 ± 6.99 nm
Skewness = -0.52
(n = 21)
Mean = 0.78 ± 0.08
(n = 21)
Mean = 59.32 ± 9.01 nm
Skewness = -1.11
(n = 26)
Mean = 44.20 ± 8.88 nm
Skewness = -0.56
(n = 26)
Mean = 0.75 ± 0.10
(n = 26)
Mean = 54.01 ± 9.88 nm
Skewness = -0.75
(n = 24)
Mean = 37.93 ± 7.20 nm
Skewness = -0.96
(n = 24)
Mean = 0.70 ± 0.08
(n = 24)
Mean = 57.02 ± 7.80 nm
Skewness = -1.08
(n = 27)
Mean = 42.56 ± 9.20 nm
Skewness = -0.61
(n = 27)
Mean = 0.74 ± 0.10
(n = 27)
Mean = 53.29 ± 8.00 nm
Skewness = -1.02
(n = 19)
Mean = 42.58 ± 9.20 nm
Skewness = -1.00
(n = 19)
Mean = 0.79 ± 0.11
(n = 19)
Mean = 58.81 ± 5.38 nm
Skewness = -0.30
(n = 26)
Mean = 43.89 ± 4.94 nm
Skewness = -0.69
(n = 26)
Mean = 0.75 ± 0.08
(n = 26)
Mean = 55.15 ± 9.56 nm
Skewness = -1.32
(n = 23)
Mean = 45.01 ± 10.09 nm
Skewness = -1.84
(n = 23)
Mean = 0.81 ± 0.12
(n = 23)

Fig. S3 Magnetosome micromorphological features of magnetotactic spirilla cells in microcosm 1. First column: TEM images of cells; second column: TEM images of magnetosome chains; third column: magnetosome distribution histograms (including magnetosome length, width, and width/length ratio).

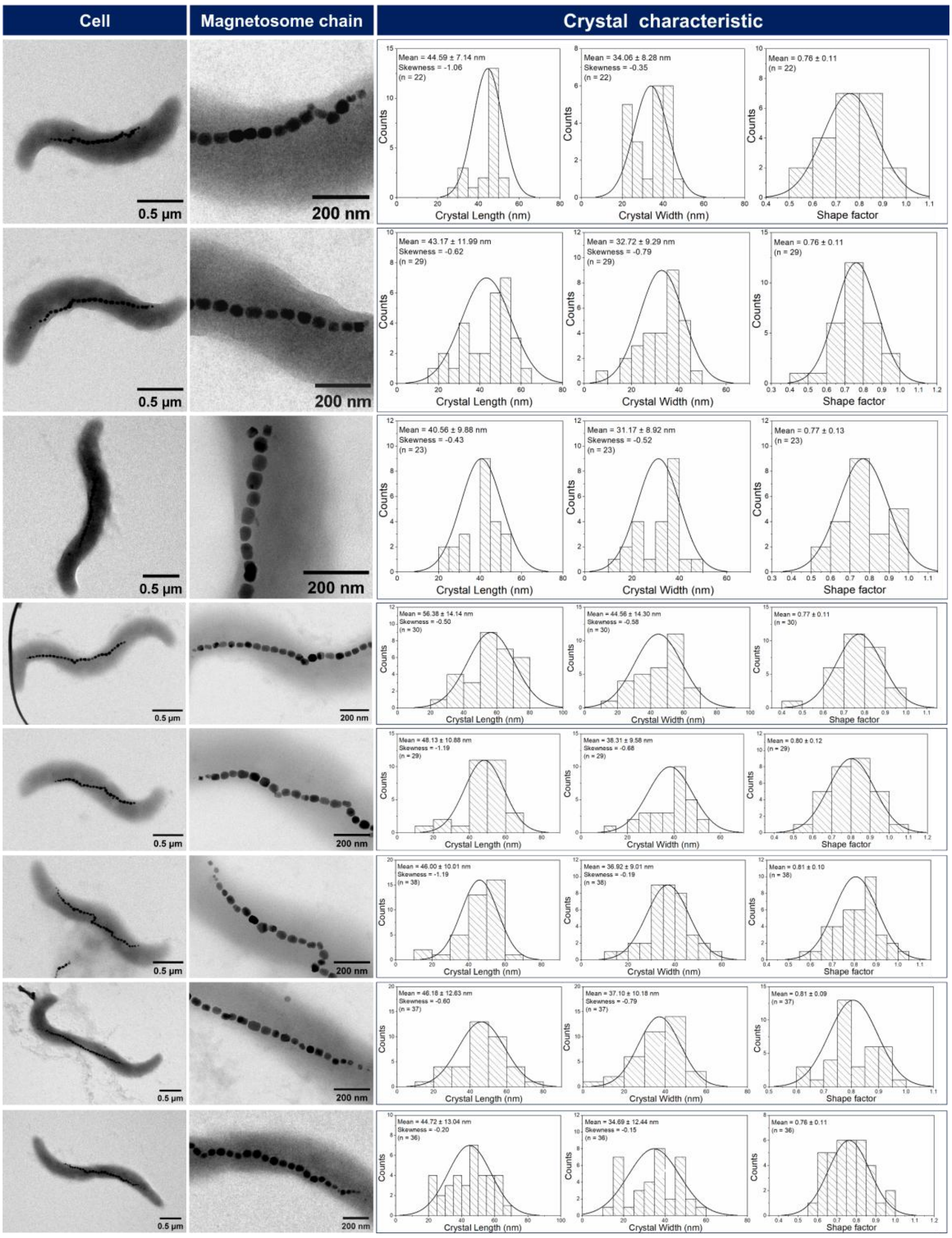

Fig. S4 Magnetosome micromorphological features of magnetotactic spirilla cells in microcosm 2. First column: TEM images of cells; second column: TEM images of magnetosome chains; third column: magnetosome distribution histograms (including magnetosome length, width, and width/length ratio).

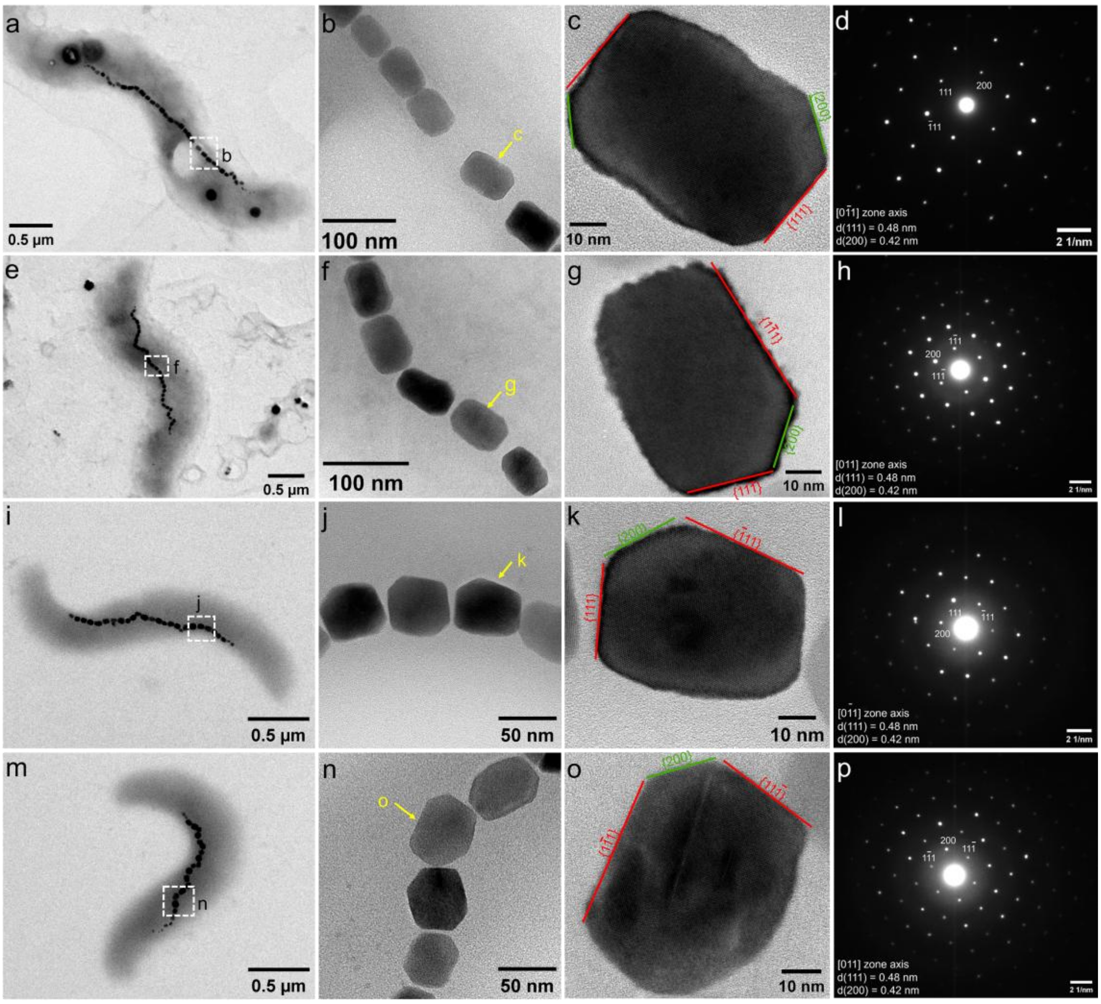

Fig. S5 Morphological characteristics of cells and magnetic particles from representative magnetotactic spirilla cells in (a–h) microcosm 1 and (i–p) microcosm 2. First column: TEM images of magnetotactic spirilla cells. Second column: zoomed-in TEM images of magnetosome chains in the white dashed boxes in column 1 images. Third column: HRTEM image of a particle (indicated by the yellow arrow in each column 2 image), with Miller indices of crystal faces indicated by solid lines. Fourth column: SAED pattern for the particle with Miller indices and calculated *d*-spacings.

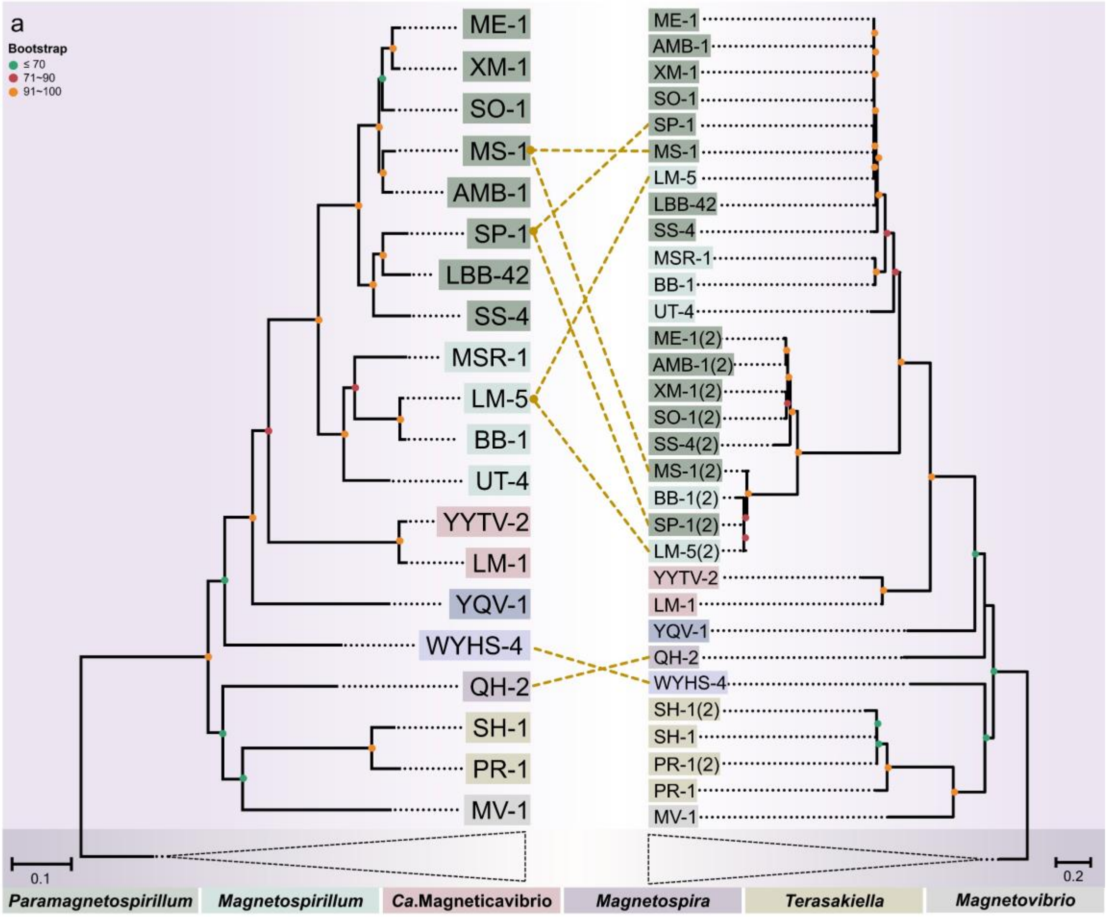

Fig. S6 Topological comparisons of alphaproteobacterial MTB phylogenetic trees based on (a) whole-genome and (b) magnetosome core gene sequences, including both *mamAB* operon and *mamAB-2* cluster. Both phylogenetic trees are constructed using the maximum likelihood method. MTB strains from other phyla are selected as the outgroup to root the tree. Bootstrap values at nodes are given as percentages of 1000 replicates. Scale bars represent sequence divergence of (a) 10% and (b) 20%, respectively. For the *mamAB-2* phylogeny, we used a conserved subset of genes (*mamE*, *mamJ*, *mamO*, *mamQ*, *mamR*, *mamB*) that are shared by ≥45% of alphaproteobacterial MTB. Strains of the same genus in the *Alphaproteobacteria* are indicated with the same color. Strains WYHS-4 and YQV-1 belong to unnamed new genera of the *Alphaproteobacteria* class. The yellow dashed line indicates inconsistent genus topology in the two phylogenetic trees.

Table S1 16S rRNA sequences retrieved from selected laboratory microcosms 1 and 2 using the 28F/1391R primer pair.

Total clone numbers	OTU/Strain	No. of clones	Percentage of clones	OTU origin	Most similar strain	Identity	Accession
49	**OTU1$_{28F}$ (YYTV-2)**	9	18.37%	Microcosm 1	*Ca.* Magneticavibrio boulderlitore LM-1	96.61%	JF490044
	**OTU2$_{28F}$ (YYTS-2)**	8	16.33%		Uncultured alpha proteobacterium clone WYH-50	98.38%	JX537788
	**OTU3$_{28F}$ (YYTS-3)**	2	4.08%		*Paramagnetospirillum* sp. B9-5-1	95.32%	FJ562217
	**OTU4$_{28F}$**	1	2.04%		*Ferribacterium limneticum*	99.78%	CP075192
	**OTU5$_{28F}$**	19	38.78%	Microcosm 2	Magnetococcales clone UR-2	98.87%	MK813937
	**OTU6$_{28F}$**	5	10.20%		*Magnetococcus* sp. clone XQGC-1	99.55%	ON340524
	**OTU7$_{28F}$ (YYTS-5)**	3	6.12%		*Paramagnetospirillum marisnigri* SP-1	96.75%	NR149242
	**OTU8$_{28F}$ (YYTS-7)**	2	4.08%		*Paramagnetospirillum marisnigri* SP-1	94.60%	NR149242

Table S2 FISH probes used in this study.

Probe name	Target group	Oligonucleotide sequence (5' to 3')	Positions	T_m (°C)	Formamide conc.	Mismatched sequence number	Reference
EUB338	Most bacteria	GCTGCCTCCCGTAGGAGT	338–355	64	35%	--	(Amann et al., 1990)
YYTV2-920	YYTV-2	AAACCATCTCTGGTAACCGCC	920-939	64	35%	0	This study
YYTS2-1106	YYTS-2	GAAGGTTCCCATCTCTAGGAA	1106-1126	62	32%	0	
YYTS3-924	YYTS-3	AGGAGCCATCTCTGACCCAC	924-943	64	35%	0	
YYTS5-917	YYTS-5	CAAATCTCTCTGGCCGGTATC	917-937	64	35%	1	
YYTS7-532	YYTS-7	CACTTCTGACTATGGTCACC	532-551	60	28%	2	

Notes: Species-specific oligonucleotide probes were designed using the offline tool DNAMAN (Version 7.0, Lynnon Biosoft, USA) and were synthesized at the Huada Genome Center (Beijing, China). The corresponding melting temperature (*T*m) was measured directly during probe synthesis, and the formamide concentration (conc.) was calculated where concentration = (T_m - 46) × 2. YYTS5-917 and YYTS7-532 probes could also match other 16S rRNA sequences and were, therefore, not strictly species-specific. Although these mismatched sequences may come from other MTB species, they were not detected in our samples. Therefore, these two probes were used in this study. For more detailed information, see Table S3.

Table S3 Mismatched information of FISH probes used in this study.

Probe name	No. of mismatched sequences	Name of mismatched sequence	Identity with target group (%)	Accession	Taxon
YYTS5-917	1	*Phaeospirillum fulvum*	96.36	D14433	*Alphaproteobacteria*
YYTS7-532	2	*Magnetospirillum* sp. Sel-1	95.43	KC252629	*Alphaproteobacteria*
		Paramagnetospirillum marisnigri	94.6	KC252630	

Notes: The specificity of both probes was evaluated using the online probe evaluation tools TestProbe of the SILVA database. Mismatched sequences were downloaded from the NCBI database. One and two 16S rRNA sequences can match probes YYTS5-917 and YYTS7-532, respectively. However, all three 16S rRNA sequences from the *Alphaproteobacteria* class were not found in this sample. This indicates that the two probes can be used in this study.

Table S4 16S rRNA sequences retrieved from selected laboratory microcosms 1 and 2 using the 27F/1492R primer pair.

Total clone number	OTU	No. of clones	Percentage of clones	Most similar strain	Identity	Accession
54	$OTU1_{27F}$	13	46.4%	*Magnetococcus* sp. clone THC-1	99.79%	MN396570
	$OTU2_{27F}$	11	39.3%	*Magnetococcus* sp. clone WYHC-1	99.45%	MN396452
	$OTU3_{27F}$	21	72.41%	Magnetococcales clone UR-2	98.89%	MK813937
	$OTU4_{27F}$	6	20.69%	*Magnetococcus* sp. clone XQGC-1	99.73%	ON340524
	$OTU5_{27F}$	1	3.45%	*Magnetococcus* sp.clone MYC-4	99.93%	MN372080
	$OTU6_{27F}$	4	14.3%	*Sphingomona*s sp. G12	99.04%	GU086447
	$OTU7_{27F}$	1	3.45%	*Achromobacter* sp. GKA-1	99.87%	EU520399

Table S5 Coverage and specificity of probe used to amplify 16S rRNA genes of *Alphaproteobacteria*.

Single primer	Oligonucleotide sequence (5 '-3')	*T*m (°C)	Coverage for the *Alphaproteobacteria* class (%)	Coverage for other bacteria phyla/classes (%)	Other matched phyla/classes	Reference
28F	ARCGAACGCTGGCGGCA	60.6	74.1	0.36	*Gammaproteobacteria*; *Hydrogenedentes*; *Verrucomicrobiota*; *Bdellovibrionota*; *Planctomycetota*; *Desulfobacterota*	Ashelford et al., 2002
528F	CGGTAATACGRAGGGRGYT	55.52	72.7	0	*Acidobacteriota*	Pfeiffer et al., 2014
689R	CBAATATCTACGAATTYCACCT	53.16	82.8	0.31	*Desulfobacterota*; *Myxococcota*; *Firmicutes*	Pfeiffer et al., 2014
27F	AGAGTTTGATCCTGGCTCAG	55.4	66.1	59.59	Most bacteria	Lane, 1991
1391R	GACGGGCGGTGWGTRCA	60.6	86	77.79	Most bacteria	Lane, 1991
1492R	GGTTACCTTGTTACGACTT	57.3	42.1	30.92	Most bacteria	Lane, 1991

Notes: The percentage coverage of each primer was calculated using the SILVA database (https://www.arb-silva.de/), which contains 381,535 full-length 16S rRNA sequences (>1400 bp) from Bacteria domain, including 33787 affiliated with the *Alphaproteobacteria* class.

Table S6 Coverage and specificity of primer pairs used to amplify 16S rRNA genes of *Alphaproteobacteria* and other phyla.

**Primer pair**	**Length (bp)**	**Coverage for the *Alphaproteobacteria* class (%)**	**Coverage for other bacteria phyla/classes (%)**	**Other matched phyla/classes**	**Reference**
27F/1492R	~1,450	24.7	23.07	Most bacteria	Lane, 1991
28F/1391R	~1350	66.1	0.49	*Gammaproteobacteria*; *Hydrogenedentes*; *Verrucomicrobiota*; *Bdellovibrionota*; *Planctomycetota*	Ashelford et al., 2002<br>Lane, 1991
28F/1492R	~1,450	20.4	0.29	*Hydrogenedentes*; *Desulfobacterota*; *Bdellovibrionota*	Ashelford et al., 2002<br>Lane, 1991
27F/1391R	~1350	61	54.79	Most bacteria	Lane, 1991
528F/1492R	~950	16.4	0.03	*Acidobacteriota*	Pfeiffer et al., 2014<br>Lane, 1991
528F/1391R	~850	64.6	0.07	*Acidobacteriota*; *Gammaproteobacteria*;	Pfeiffer et al., 2014<br>Lane, 1991
28F/689R	~650	64.7	0.01	SAR324 clade,	Ashelford et al., 2002<br>Pfeiffer et al., 2014
27F/689R	~650	54.6	0.24	*Desulfobacterota*; *Firmicutes*; *Nitrospirota*	Lane, 1991<br>Pfeiffer et al., 2014

Notes: The percentage coverage of each primer pair was calculated using the SILVA database (https://www.arb-silva.de/), which contains 381,535 full-length 16S rRNA sequences (>1400 bp) from Bacteria domain, including 33787 affiliated with the *Alphaproteobacteria* class.

Table S7 TEM morphology data for each spirilla MTB cell in microcosms 1 and 2.

Microcosm	Strain number	Cell			Magnetosome			
		Cell length (μm)	Cell width (μm)	Shape	Particle number	Average width (nm)	Crystal morphology	Average shape factor
Microcosm 1	43	3.42	0.8	Spirillum	33	41.78	Elongated cubo-octahedral	0.74
		2.4	0.55		24	40.13		0.71
		2.84	0.62		26	41.6		0.74
		2.98	0.72		21	47.52		0.78
		3.77	0.65		26	44.2		0.75
		3.38	0.65		24	37.93		0.7
		2.91	0.75		27	42.56		0.74
		3.37	0.72		19	42.58		0.79
		2.91	0.85		26	41.95		0.71
		2.89	0.73		23	45.01		0.81
		3.8	0.77		32	45.03		0.8
		3.03	0.72		27	43.56		0.73
		2.89	0.68		29	40.58		0.74
		3.08	0.76		27	39.24		0.71
		3.273	0.79		31	42.41		0.72
		3.09	0.72		30	44.56		0.77
		3.07	0.73		35	40.43		0.71
		2.341	0.57		32	42.39		0.74
		3.225	0.831		31	42.56		0.74
		3.27	0.76		25	43.37		0.71
		2.67	0.67		26	47.42		0.82
		3.51	0.87		39	40		0.7
		3.51	0.65		36	44.26		0.79
		2.32	0.65		21	41.88		0.74
		2.52	0.65		29	38.97		0.69
		3.38	0.65		27	44.16		0.78
		2.82	0.67		31	43.65		0.72
		3.83	0.75		29	40.62		0.72
		2.96	0.59		27	43.29		0.77
		3.81	0.8		29	42.2		0.75
		2.78	0.65		25	41		0.7
		3.08	0.65		23	39.48		0.68
		2.57	0.79		30	44.33		0.77
		2.51	0.71		30	41.16		0.75
		3.29	0.74		31	45.78		0.77
		3.42	0.8		34	40.34		0.72
		2.92	0.75		24	43.56		0.8
		2.25	0.63		20	43.85		0.8
		2.73	0.54		18	41.56		0.71
		3.79	0.76		20	40.11		0.7
		3.01	0.74		30	41.86		0.7
		2.57	0.64		23	37.6		0.7
		3.05	0.7		30	41.49		0.71
Microcosm 2	9	2.61	0.39	Spirillum	24	36.17	Elongated cubo-octahedral	0.79
		1.86	0.34		22	34.06		0.76
		2.08	0.33		29	32.72		0.76
		2.46	0.37		29	38.31		0.8
		2.69	0.37		38	36.92		0.81
		3.13	0.36		37	37.1		0.81
		3.3	0.35		36	34.69		0.76
		2.4	0.34		23	31.17		0.77
		2.75	0.37		30	41.18		0.78

Notes: TEM images of cells and magnetosomes of spirilla MTB cells in microcosms 1 and 2 are shown in Figs. S3 and S4.

Table S8 Reported data on cellular and magnetosomal morphology characteristics of the alphaproteobacterial MTB.

Strain	Taxonomy	Accession	Cell				Magnetosome				Reference
			Shape	Length (μm)	Width (μm)	Number	Average width (nm)	Average length (nm)	Shape factor	Crystal morphology	
AMB-1		AP007255		3	0.5	15	45.00	52.94	0.85		Matsunaga et al., 2005
XM-1		KP966105		2.5	0.445	10	37.15	43.70	0.85		Wang et al., 2015
MS-1		M58171		3	0.3	18	45.00	50.00	0.9		Smalley et al., 2015
SO-1	*Paramagnetospirillum*	NR_149241		2.15	0.3	18	31.77	35.30	0.9		Dziuba et al., 2016
LBB-42		MH571849		2.7	0.5	15	53.34	59.93	0.89		Koziaeva et al., 2019
SP-1		NR_149242	Spirillum	1.45	0.35	17	29.69	33.36	0.89	Cubo-octahedral	Dziuba et al., 2016
SS-4		JF490045		2.74	0.58	21	41.89	55.12	0.76		Monteil et al., 2020
MSR-1		NR_121771		3.193	0.464	20	42.00	44.21	0.95		Wang et al., 2014
LM-5	*Magnetospirillum*	JF490040		3.3	0.48	21	43.42	51.08	0.85		Monteil et al., 2020
BB-1		NR_149243		3	0.3	12	30.50	32.80	0.93		Dziuba et al., 2016
**YYTV-2***	*Ca.* Magneticavibrio	JBHMJB000000000	Vibrio	2.39	0.75	22	41.20	56.44	0.73	Elongated cubo-octahedral	This study
LM-1		NR_149243		2.252	0.353	17	38.44	49.28	0.78	Prismatic	Lefèvre et al., 2012
CCP-1	NA	MT021453	Rod	2.78	0.96	14	40.00	57.97	0.69	Truncated octahedral	Monteil et al., 2021
WYHS-1	NA	MW646017	Spirillum	3.2	1.4	12	32.80	34.53	0.95		Li et al., 2021
YQV-1	*Azospirillum*	ON340537	Vibrio	2.5	0.8	19	82.00	88.17	0.93		Liu et al., 2023
XQGS-1		KJ442651	Spirillum	2.43	0.84	17	83.30	88.30	0.94		Liu et al., 2021
QH-2	*Magnetospira*	KJ442651	Spirillum	2	0.8	16	58.00	81.69	0.71	Prismatic	Ji et al., 2013
MMS-1		EU861390		2	0.3	17	52.82	65.21	0.81		Williams et al., 2012
MV-1	*Magnetovibrio*	NR_118660	Vibrio	1.5	0.3	10	35.00	55.56	0.63		Bazylinski et al., 2013
SH-1	*Terasakiella*	NZ_CP038255	Spirillum	2.886	0.508	12	35.70	48.24	0.74		Du et al., 2019
PR-1		KJ442651		1.6	0.5	14	34.00	44.16	0.77		Monteil et al., 2018
WYHS-4	NA	NR_149243		2.2	0.6	21	43.00	75.44	0.57		Liu et al., 2023

Notes: Name in bold with * are novel strains identified morphologically with genome obtained.
